# Competing effects of soil fertility and toxicity on tropical greening

**DOI:** 10.1038/s41598-020-63589-1

**Published:** 2020-04-21

**Authors:** Joshua B. Fisher, Naga V. Perakalapudi, Benjamin L. Turner, David S. Schimel, Daniela F. Cusack

**Affiliations:** 1 Jet Propulsion Laboratory, California Institute of Technology, 4800 Oak Grove Drive, Pasadena, CA 91109 USA; 2 Department of Astronautical Engineering, University of Southern California, 854 Downey Way, Los Angeles, CA 90089 USA; 3 Smithsonian Tropical Research Institute, Apartado 0843-03092 Balboa, Ancon Panama; 4 Department of Ecosystem Science and Sustainability, Colorado State University, Campus Delivery 1476, Fort Collins, CO 80523 USA

**Keywords:** Tropical ecology, Tropical ecology

## Abstract

Tropical forests are expected to green up with increasing atmospheric CO_2_ concentrations, but primary productivity may be limited by soil nutrient availability. However, rarely have canopy-scale measurements been assessed against soil measurements in the tropics. Here, we sought to assess remotely sensed canopy greenness against steep soil nutrient gradients across 50 1-ha mature forest plots in Panama. Contrary to expectations, increases in *in situ* extractable soil phosphorus (P) and base cations (K, Mg) corresponded to declines in remotely sensed mean annual canopy greenness (r^2^ = 0.77–0.85; p < 0.1), controlling for precipitation. The reason for this inverse relationship appears to be that litterfall also increased with increasing soil P and cation availability (r^2^ = 0.88–0.98; p < 0.1), resulting in a decline in greenness with increasing annual litterfall (r^2^ = 0.94; p < 0.1). As such, greater soil nutrient availability corresponded to greater leaf turnover, resulting in decreased greenness. However, these decreases in greenness with increasing soil P and cations were countered by increases in greenness with increasing soil nitrogen (N) (r^2^ = 0.14; p < 0.1), which had no significant relationship with litterfall, likely reflecting a direct effect of soil N on leaf chlorophyll content, but not on litterfall rates. In addition, greenness increased with extractable soil aluminum (Al) (r^2^ = 0.97; p < 0.1), but Al had no significant relationship with litterfall, suggesting a physiological adaptation of plants to high levels of toxic metals. Thus, spatial gradients in canopy greenness are not necessarily positive indicators of soil nutrient scarcity. Using a novel remote sensing index of canopy greenness limitation, we assessed how observed greenness compares with potential greenness. We found a strong relationship with soil N only (r^2^ = 0.65; p < 0.1), suggesting that tropical canopy greenness in Panama is predominantly limited by soil N, even if plant productivity (e.g., litterfall) responds to rock-derived nutrients. Moreover, greenness limitation was also significantly correlated with fine root biomass and soil carbon stocks (r^2^ = 0.62–0.71; p < 0.1), suggesting a feedback from soil N to canopy greenness to soil carbon storage. Overall, these data point to the potential utility of a remote sensing product for assessing belowground properties in tropical ecosystems.

## Introduction

Tropical forests are the ‘lungs’ of our planet, absorbing and storing the largest carbon stocks of all terrestrial ecosystems^1–4^. They are also the source of among the largest uncertainties to projections of Earth’s climate, due to their poorly understood responses to changes in CO_2_, climate, land use, and nutrient cycling^5–9^. Numerous modeling studies have suggested that tropical forest productivity is increasing^5,6^, and present day observational indicators from space suggest that the tropics are greening likely due to CO_2_ fertilization, in the face of increasing pressure from droughts and disturbance^10–16^.

Despite the apparent greening of the tropics, it is well-known that there is widespread nutrient limitation to net primary productivity^17–23^. The extent, nature, and impacts of soil nutrient availability are debated, however. Some studies show strong tropical forest responses to phosphorus (P)^24–30^, others to nitrogen (N)^31–33^, some indicate strong influence of multiple macro-nutrients on tropical forest processes^17,22,34–36^, and others suggest that micronutrients and/or base cations drive key ecosystem carbon processes^37–43^. These studies have also linked soil nutrients to a wide range of different tropical forest carbon cycle responses, including litterfall rates, basal growth, root production, and soil decomposition, for example, with responses varying by tree age and size^20,24,30,37,44,45^.

The reason for such different results is that tropical ecosystems are both extremely diverse and challenging to work in, such that ground data at specific points cannot adequately capture the dynamics of variation across the biome as a whole^1,21,30,46^. Remote sensing data, on the other hand, have the potential to scale across landscapes, but lack insight from ground-scale controls over canopy properties^1,9^. Moreover, studies of remote sensing observations and those of *in situ* soil measurements have been done largely in isolation of one another. As such, there exists a critical gap in our understanding of how soil properties manifest in canopy-scale properties across tropical landscapes.

We are currently in a golden age of satellite remote sensing of terrestrial vegetation. Decades of work with Landsat, AVHRR, and MODIS have yielded a wealth of data and insights across multiple properties from albedo and surface temperature to greenness, as well as a host of higher-order datasets for productivity, evapotranspiration, nutrient limitation, and mycorrhizal association, for example^19,47–51^. Although the potential is large for ecosystem assessment of soil nutrient linkages to canopy properties with these data, these remote sensing data products have not been partnered with a similarly large-scale soils database from the tropics.

One of the most comprehensive tropical soils datasets to date has been collected over a decade by the Smithsonian Tropical Research Institute (STRI) across 50 1-ha plots in lowland tropical forest sites across the Isthmus of Panama, covering a wide range in geology, climate, and biodiversity^43,52–54^. The strong diversity of environmental conditions across the study area makes observed trends broadly applicable to much larger geographical ranges, and the relatively large-scale of the plots makes this dataset compatible with remote sensing attributes. Particularly characteristic to these sites is that, although they fall along a distinct precipitation gradient, the diverse geology drives variability in soil nutrients such that they are only weakly or not at all correlated with precipitation, making this dataset uniquely suited for disentangling effects of precipitation from soil properties. The STRI dataset is an ideal source with which to combine terrestrial vegetation remote sensing products to assess below- and above-ground coupling, and soil nutrient controls on canopy-scale properties.

The objective of this study was to assess remotely sensed canopy properties, particularly gradients of greenness and derived products, across a large-scale tropical gradient in soil nutrient availability and other chemical properties. We tested predictions that soil nutrients and soil carbon stocks would be positively related to canopy properties related to productivity, such as greenness. Such relationships would reflect the control of soil nutrients over plant productivity, and subsequent control of plants over carbon inputs to ecosystems. The overall approach was to enable identification of potential couplings between belowground and aboveground measurements, disentangling effects of precipitation from soil properties.

## Methods

### Field measurements

All plots were in lowland tropical forests (elevation 10–410 m above sea level) and included old growth primary and mature secondary forests across a precipitation gradient in Panama^55^. The climate is tropical monsoon, with a mean annual temperature of 26 °C and mean monthly temperature variation of <1 °C during the year^52,55,56^. The wetter Caribbean coast receives 4000 mm y^−1^ MAP and has a shorter dry season (~115 days) compared with the drier Pacific coast, which receives 1750 mm y^−1^ MAP and has a longer dry season (~150 days). Meteorological data were measured at each site. Radiation is inversely proportional to precipitation;^57–59^ and, given low temperature variability, we assessed each site for precipitation normalization.

Soils were collected during the 8-month wet season as described in Turner, *et al*.^60^. Soil properties, litterfall, and aboveground biomass were measured using standard protocols, as previously reported^30,43,52–55^. In brief, total soil C, N, P, resin-extractable P, extractable base cations, pH, soil texture, root biomass, and bulk density were measured to 1 m depth in 1-ha plots at each site (Supplementary Fig. 1) sampling both across the spatial variation within plots, and in soil pits (1.8 m deep) outside the edge of each plot.

The soils developed on a range of geological substrates^54,55,61,62^, including volcanic (basalt, andesite, agglomerate, rhyolitic tuff) and marine sedimentary (limestone, calcareous sandstone, siltstone, mudstone) lithologies. As a result, soils have marked variation in fertility^53,54^ and soil order, including Inceptisols, Mollisols, Alfisols, Ultisols, and Oxisols. Soil nutrients such as P spanned high and low fertility across multiple soil types. Statistics are shown here by soil order across plots except for N, P, Ca, and Zn.

Aboveground dry biomass (AGB) for all trees >10 cm in diameter at breast height (DBH) was estimated in each of the plots using allometric equations. Methodological details and examination of errors have been published^63^, with the most recent DBH decadal census used for this paper^30^. Litterfall biomass was collected at a subset of 8 sites biweekly for one year as described in Cusack, *et al*.^43^. All soils, AGB, and litterfall data are published as an online supplementary file in Cusack, *et al*.^43^. There were no significant differences among plots for aboveground biomass, canopy cover, species composition, and successional stage (all mature trees).

### Remote sensing

We processed and analyzed data from 10 instruments on 8 different satellites, yielding a total of >70 different data products (Supplementary Fig. 1). The instruments and satellites included: Enhanced Thematic Mapper Plus (ETM+), Operational Land Imager (OLI), and Thermal Infrared Sensor (TIRS) on Landsat 7 and 8; MODerate resolution Imaging Spectroradiometer (MODIS) on Terra and Aqua; radar and radiometer on Soil Moisture Active Passive (SMAP); three high-resolution spectrometers on the Orbiting Carbon Observatory 2 (OCO-2); Geoscience Laser Altimeter System (GLAS) on Ice, Cloud, and land Elevation Satellite (ICESat); and, Hyperion on Earth Observing-1 (EO-1). Data were processed for an annual summary statistic for the most recent year (2016), except where otherwise available (e.g., ICESat, 2005).

#### Landsat 7 and 8: ETM+, OLI, TIRS

ETM+, OLI, and TIRS on Landsat 7 and 8 recorded measurements in 11 spectral bands (0.43–12.51 μm). We processed and analyzed data products for Normalized Difference Vegetation Index (NDVI), Enhanced Vegetation Index (EVI), Albedo, and Land Surface Temperature (LST)^64^. We acquired the data from the United States Geological Survey (USGS) Earth Resources Observation and Science (EROS) Science Processing Architecture (ESPA) for both Landsat 7 (LE07, L1TP) and Landsat 8 (LE08, L1TP). Data were screened and masked for clouds based on the provided metadata and quality flags. Albedo for Landsat 7 was calculated through Top of Atmosphere (ToA) from bands 1, 3, 4, 5, and 7; and, for Landsat 8 from bands 2, 4, 5, 6, and 7 following Liang^65^. LST for Landsat 7 was calculated through surface reflectance from band 6; and, for Landsat 8 from band 10. Surface reflectance was converted to LST following: ${L}_{\lambda }={M}_{L}{Q}_{cal}+{A}_{L}$, where *L*_*λ*_ is ToA spectral radiance (W m^−2^ srad^−1^ μm^−1^), *M*_*L*_ is a band-specific multiplicative rescaling factor from the metadata, *A*_*L*_ is a band-specific additive rescaling factor from the metadata, and ${Q}_{cal}$ is quantized and calibrated standard product pixel value^66^. ToA brightness temperature (*T*, K) is: $T=\,\frac{{K}_{2}}{\log \left(\frac{{K}_{1}}{{L}_{\lambda }}+1\right)}$, where *K*_1_ and *K*_2_ are band-specific thermal conversion constants from the metadata. All Landsat products were available at 16-day time steps and 30 m spatial resolution.

#### Terra and Aqua: MODIS

MODIS on Terra and Aqua recorded measurements in 36 spectral bands (620 nm–965 nm and 3.66 μm–14.385 μm). We processed and analyzed data products for NDVI and EVI (MOD13Q1 V005, MYD13Q1 V005), gross primary productivity (GPP) (MOD17A2 V005, MOD17A3 V055, MYD17A2 V005), net primary productivity (NPP) (MOD17A3 V055, MYD17A3H V006), evapotranspiration (ET) (MOD16A2 V006), Leaf Area Index (LAI) and Fraction of Absorbed Photosynthetically Active Radiation (FAPAR) (MCD15AC3H V006), LST Day and Night (MOD11A1 V006, MYD11A1 V006), and Albedo Bands 1–10 Day and Night (MCD43A3 V005)^67^. Three ground sites were excluded from analyses of evapotranspiration due to pixel contamination from adjacency to open water (Plot 1: ocean; Plots 25 & 26: canal). We acquired the data from the USGS EROS Earth Observing System Data and Information System (EOSDIS) Land Processes DAAC for tiles h09v08 and h10v08. Data were screened and masked for clouds based on the provided metadata and quality flags. MODIS NDVI and EVI were available from daily measurements at 16 day temporal composites and 250 m spatial resolution; LAI, FAPAR, and Albedo at 4 day composites (LAI, FAPAR) or 16 day composites (Albedo) at 500 m; and, GPP, NPP, ET, and LST at 8 day composites (GPP, NPP, ET) or daily composites (LST) at 1 km. MODIS NPP and ET products were combined to produce the Canopy Greenness Limitation product described in Fisher, *et al*.^19^. Canopy greenness limitation (N.L.) is calculated from the ratio of NDVI to evapotranspiration (AET) normalized to a percentage, increasing further from the upper bound in the global scatterplot of average maximum paired AET and NDVI:

$$N.L.=\frac{paire{d}_{max}\left(\frac{NDV{I}_{x}}{AE{T}_{x}}\right)-paire{d}_{max}\left(\frac{NDV{I}_{min}}{AE{T}_{min}}\right)}{paire{d}_{max}\left(\frac{NDV{I}_{max}}{AE{T}_{max}}\right)-paire{d}_{max}\left(\frac{NDV{I}_{min}}{AE{T}_{min}}\right)}$$

where the paired_max_ is for the quotient; the x subscript is for the given pixel; and the min and max are for across the entire data set to apply consistent global normalization.

#### SMAP: radar and radiometer

The SMAP radiometer recorded measurements at 1.41 Hz, and the radar at 1.26 Hz. We processed and analyzed data products for Soil Moisture (SM) and Albedo (SP3SMP V004, SPL3SMP E V001); Surface Soil Moisture (SSM), Root Zone Soil Moisture (RZSM), and LAI (SPL4SMGP V002); and, Net Ecosystem Exchange (NEE), Heterotrophic Respiration (Rh), Soil Organic Carbon (SOC), and GPP (SPL4CMDL V002)^68^. We acquired the data from the NASA Distributed Active Archive Center (DAAC) at the National Snow and Ice Data Center (NSIDC). SMAP data bins were extracted based on latitude and longitude from the EASE Grid projection and processed to WGS84 projection. Recommended retrieval quality flags were applied. Radar data were available only for 2015Q1-2. All SMAP products were available at daily time steps with SSM, RZSM, and LAI at 3 hourly time steps and 9 km spatial resolution; SM and Albedo were additionally available at 36 km (radiometer).

#### OCO-2: High-Resolution Spectrometers

The high-resolution spectrometers on OCO-2 recorded measurements at 0.76, 1.61 and 2.06 μm. We processed and analyzed data products for SIF generated from the Iterative Maximum a Posteriori Differential Optical Absorption Spectroscopy (IMAP-DOAS) preprocessor (L2IDP)^69^. We acquired the data from https://oco2.gesdisc.eosdis.nasa.gov/data/. OCO-2 data bins were extracted based on latitude and longitude from the EASE Grid projection and processed to WGS84 projection. Data were aggregated to monthly means for data/bands SIF757 and SIF771 following Sun, *et al*.^70^. OCO-2 SIF data were available at 16-day time steps and 36 km spatial resolution.

#### ICESat: GLAS

GLAS on ICESat recorded measurements from a full waveform LiDAR at 1.064 μm and 40 Hz. We processed and analyzed the vegetation canopy height product developed by Simard, *et al*.^71^, which fused GLAS with MODIS and climate data to produce the product. We acquired the data using the Spatial Data Access Tool (SDAT) from the Oak Ridge National Laboratory (ORNL) DAAC. Data were provided in GeoTIFF format, pre-screened and quality controlled. ICESat canopy height data were available as a static map from 2005 at 1 km spatial resolution.

#### EO-1: Hyperion

Hyperion on EO-1 recorded measurements in 220 spectral bands ranging between 0.4–2.5 μm (SWIR and VNIR)^72^. We acquired the L1T data (systematically terrain-corrected) from Earth Explorer. Data were provided in GeoTIFF format. Forty-three bands were removed from the Hyperion cube as uncalibrated. Cloud screening was performed with bands 31, 51, and 133 using information on Julian day, sun elevation angle (cos θ_s_), scaling factor, irradiance value (ESUN_λ_), and Earth-Sun distance (d) from the metadata. Cloud-corrected radiance was converted to reflectance (${\rho }_{p}$) using ${\rho }_{p}=\frac{\pi {L}_{\lambda }{d}^{2}}{ESU{N}_{\lambda }{\cos }_{\theta s}}$, where *L*_*λ*_ is spectral radiance at the sensor’s aperture. Hyperion data were processed from images collected over 2002–2012 at 30 m spatial resolution.

## Results and Discussion

**Figure 1:**
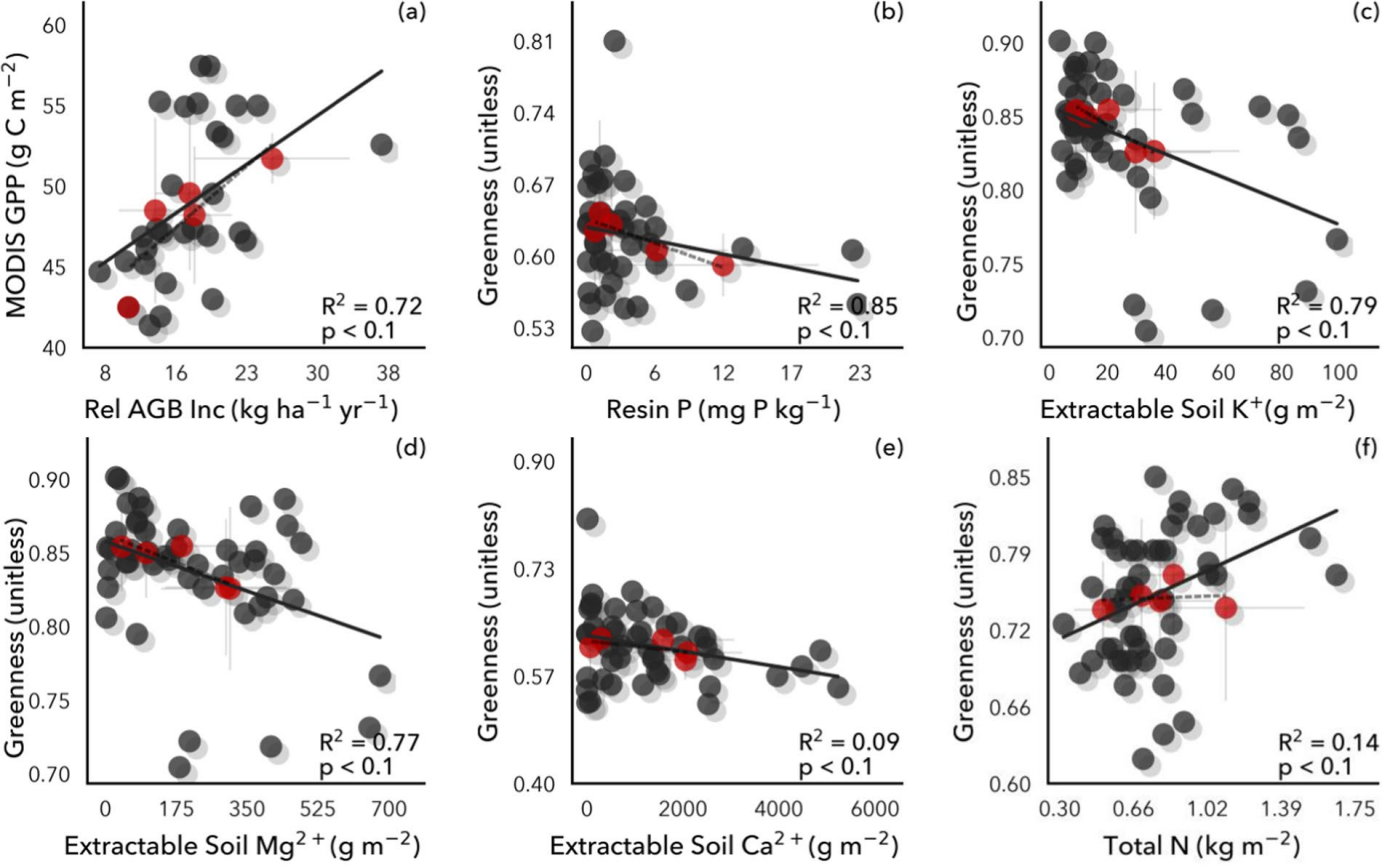
Remotely sensed canopy greenness was negatively correlated with *in situ* soil phosphorus (P) and cations (K, Mg, Ca), but positively correlated with *in situ* soil nitrogen (N) and aboveground biomass (AGB) increase. Individual plots (gray points, solid line) are aggregated by soil order (red points, dashed line; statistics shown).

**Figure 2:**
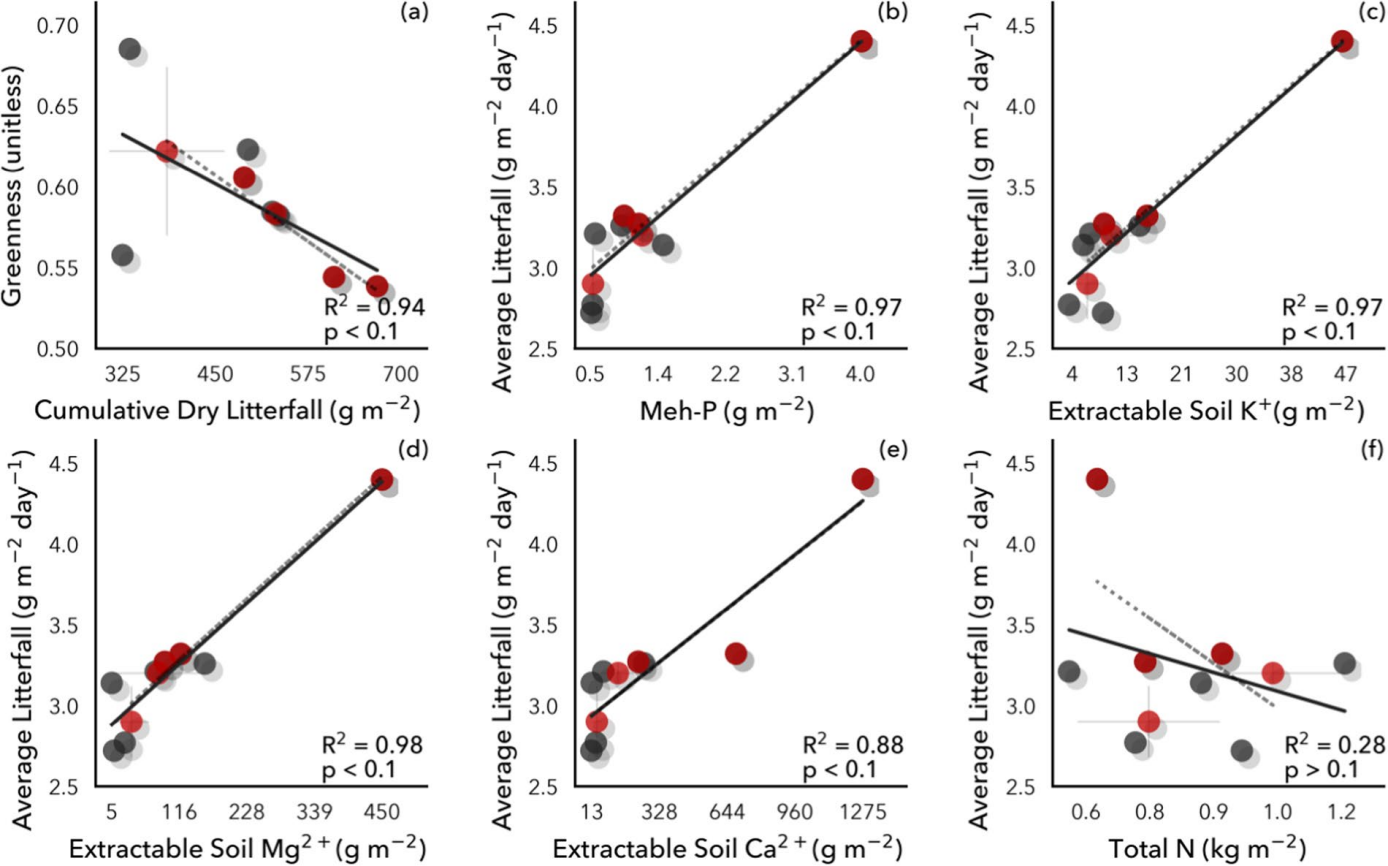
Litterfall was negatively correlated with remotely sensed canopy greenness, positively correlated with soil phosphorus (P) and cations (K, Mg, Ca), and had no relationship with soil nitrogen (N).

**Figure 3:**
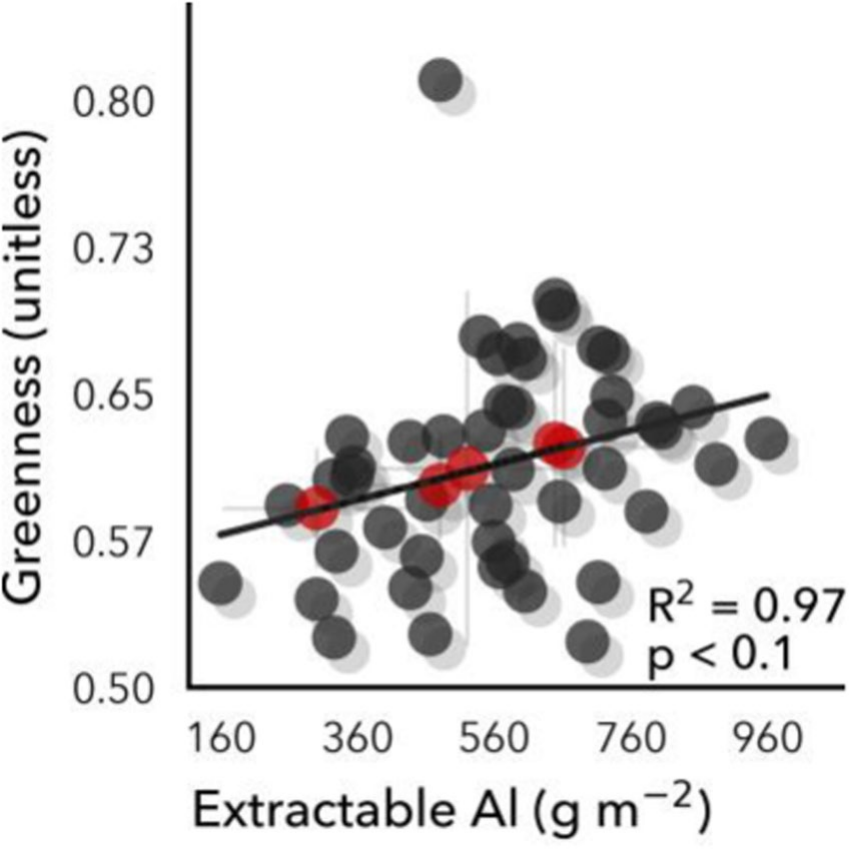
Remotely sensed canopy greenness was positively correlated with *in situ* extractable soil aluminum (Al), paradoxically given that Al is toxic to plants. It may be that tropical plants that have adapted to excess Al soils are relatively more productive than plants on less toxic soils because a significant amount of photosynthate is required to filter out the Al.

**Figure 4:**
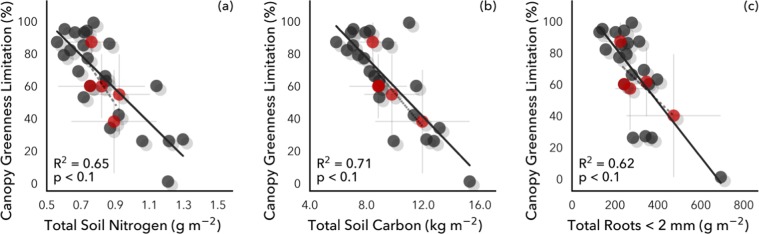
Remotely sensed canopy greenness limitation was strongly correlated with *in situ* (**a**) soil nitrogen, (**b**) soil carbon, and (**c**) total roots.

We evaluated spatial gradients of *in situ* soil nutrients and chemical properties against remotely sensed canopy properties. Remotely sensed greenness and derived products such as GPP were correlated with *in situ* measurements of relative aboveground biomass increase (r^2^ = 0.72; p < 0.1) (Fig. 1a). Contrary to expectations, as *in situ* extractable soil phosphorus (P) and base cations (calcium, Ca; potassium, K; magnesium, Mg) increased in availability across sites, remotely sensed mean annual canopy greenness decreased (r^2^ = 0.09–0.79; p < 0.1) (Fig. 1b–e), controlling for climatic differences such as precipitation (Supplementary Fig. 2). Base cations (K and Mg) were strongly negatively correlated with NDVI (Landsat 8) (r^2^ = 0.79 and 0.77), while Ca was weakly negatively correlated (r^2^ = 0.09). We use the term “Greenness” instead of NDVI in the figures to facilitate translation to science communities unfamiliar with the remote sensing terminology. All statistics for Figs.1–4 can be found in Supplementary Table 1. Assessing only the wet season, the negative relationship between canopy greenness and Ca increased in strength (r^2^ = 0.91), and remained similar for P, K, and Mg (Supplementary Fig. 2). It may be that these results could be even stronger than shown due to greenness saturation causing large point spreads at high values^73,74^.

The inverse relationship between soil P and cations with canopy greenness likely results from a strong positive relationship between annual litterfall and soil P and cations (r^2^ = 0.88–0.98; p < 0.1), indicating greater leaf turnover, and potentially shorter leaf lifespan, where soil nutrients are plentiful (Fig. 2b–f). Strong links between litterfall rates and soil fertility have been shown throughout the tropics, including Panama^20,37,75–77^. Accordingly, increasing litterfall was related to a strong decline in greenness (r^2^ = 0.94; p < 0.1) (Fig. 2a). That is, litterfall increased with soil P and cation availability across the subset of sites with litterfall measurements, helping explain the broader negative relationship between canopy greenness and soil P and cations across all sites, as an increase in litterfall can lead to a brighter surface due to fewer canopy leaves^78,79^. At the same time, these decreases in greenness with increasing soil P and cations were countered by increases in greenness with increasing soil nitrogen (N) (r^2^ = 0.14; p < 0.1) (Fig. 1f), with soil N not significantly correlated with litterfall (Fig. 2f), nor with soil P or cations.

Notably, we also found that canopy greenness was strongly positively correlated with extractable soil aluminum (Al) across soil orders (r^2^ = 0.97; p < 0.1) (Fig. 3), which initially appears paradoxical as Al is toxic to plants^80–85^. Al was not significantly correlated with soil base cations nor with litterfall. In acidic soils, Al is solubized and can be a major factor limiting plant production^86^, with increased Al concentrations in leaves associated with decreased photosynthetic rates and decreased foliar N, P, and other nutrients^87^. Extractable soil Al and manganese (Mn) were recently identified as the strongest constraints on canopy tree diameter growth for one of our Panama sites at mid-precipitation^88^. Aluminum toxicity can elicit plant responses in cell walls, plasma membranes, and symplasm^89^, and metabolic changes like autophagy as defense responses^90^, which can help repair plant tissue damages from Al toxicity. Thus, under elevated Al toxicity, plants may invest substantially in leaf repair, which could be related to longer leaf lifespans, changes in pigmentation, and/or leaf structural changes that could affect canopy reflectance properties. Thus, canopy greenness relationships with soil Al may result from change in leaf greenness not related to increased photosynthesis and growth, which canopy greenness is usually taken to indicate.

Spatial gradients in nutrients and greenness are not necessarily indicative of nutrient limitation^91^. Using a novel remote sensing index of canopy greenness limitation, we assessed how observed greenness compares with potential greenness based on energy and water availability at a broad scale^19^. We found a strong relationship with soil N only (r^2^ = 0.65; p < 0.1) (Fig. 4a), suggesting that canopy greenness in tropical forests in Panama is predominantly driven by soil N, but not by other nutrients^23,76^. This could reflect the strong association between foliar N and chlorophyll content, with plants taking up additional N even in N-rich soils (“luxury consumption”). Also, total soil C (r^2^ = 0.71; p < 0.1) and root biomass (r^2^ = 0.62; p < 0.1) were strongly correlated with the remotely sensed canopy greenness limitation product (Fig. 4b,c), such that as the canopy approached maximum theoretical greenness, soil C and root biomass also increased. This relationship with soil C stocks could reflect greater root biomass and greater plant photosynthetic activity at higher soil N, both of which could contribute to soil C stocks^92–94^. This result contrasts with a fertilization experiment at one Panama site where added N alone had no effect on litterfall or stem growth rates, although N and P added together did appear to elevate productivity^77^. The links among soil N, canopy greenness, roots, and soil C may suggest a previously unmeasured input of C to soil via root exudates, which can be a significant sink of annual photosynthate, but are challenging to measure *in situ*^95^. The strong coupling between canopy and soil properties underscores the potential utility of remote sensing for assessing belowground properties in tropical ecosystems.

Our analysis thus far has aimed at exploring first-order relationships between soil properties and canopy greenness. Although this is just a first step, one may wish to see how these results can be integrated into mechanistic, process-based modeling systems, such as terrestrial biosphere models (TBMs) or Earth system models (ESMs)^96^. The need for nutrient processes, especially in the tropics, is well-stated^27,96–99^. Certainly, building these relationships into these types of models is beyond the scope of this paper. Still, our results provide comparative behaviors for the results of future model developments^100,101^. Numerous multi-variate statistical approaches can also assign weighting to the different controls on canopy greenness responses. These approaches include, for example, multi-variate regression, artificial neural networks, random forest, principal components analysis, and structural equation modeling. We have used these approaches extensively in previous analyses^102–104^. In this analysis, however, the data distribution statistical requirements for these approaches were not always satisfied, and, while the results largely reinforced what we already found, they often minimized interesting relationships with the micronutrients. Moreover, some approaches, such as structural equation modeling, require certain assumptions about fixed pathways, or known relationships, that must be defined. Yet, our results showed unexpected responses, such as decreasing greenness with increasing P and base cations. Ultimately, soil N was the only nutrient-based significant predictor of canopy greenness limitation.

Although it had already been established that the sites were situated such that soil properties were only weakly or not at all correlated with precipitation^43,52–54^, we took additional measures to separate the influence of precipitation from soil properties on canopy characteristics^105–107^. First, we evaluated all data normalized by mean annual precipitation (Supplementary Fig. 2). Second, we assessed only wet season canopy properties from the remote sensing data (and *in situ* data, where appropriate; i.e., litterfall), though there were fewer cloud-free remote sensing data in the wet season-only analysis (Supplementary Figs. 3 and 4). This precaution avoids results driven by the large litterfall that occurs across these semi-deciduous forests during the dry season. Ultimately, the patterns and statistics remained largely unaffected by normalization to precipitation (i.e., statistically significant at p < 0.1), with correlation coefficients decreasing in some cases, but increasing in others. These steps were taken to ensure that we assessed soil effects on canopy properties independent from precipitation effects.

We assessed a large suite of soil variables and remote sensing products. Ultimately, the clearest results for the greenness analyses came from Landsat 8, which has the highest spatial resolution of all the products assessed. The high spatial resolution likely contributed to the ability to detect relatively clear patterns^107^. Though that is not to say that an improvement in spatial resolution for SIF or soil moisture would necessarily translate into stronger correlations; but, we had no ready way to test this^108^. We also tested the soil variables against MODIS NDVI, which has a coarser spatial resolution, but finer temporal resolution, than Landsat. Spatial resolution resulted in larger importance than temporal resolution in this particular analysis. This was likely due to the large spatial heterogeneity in soil properties in conjunction with the fortuitous acquisition of a sufficient number of cloud-free images from Landsat.

**Figure 5:**
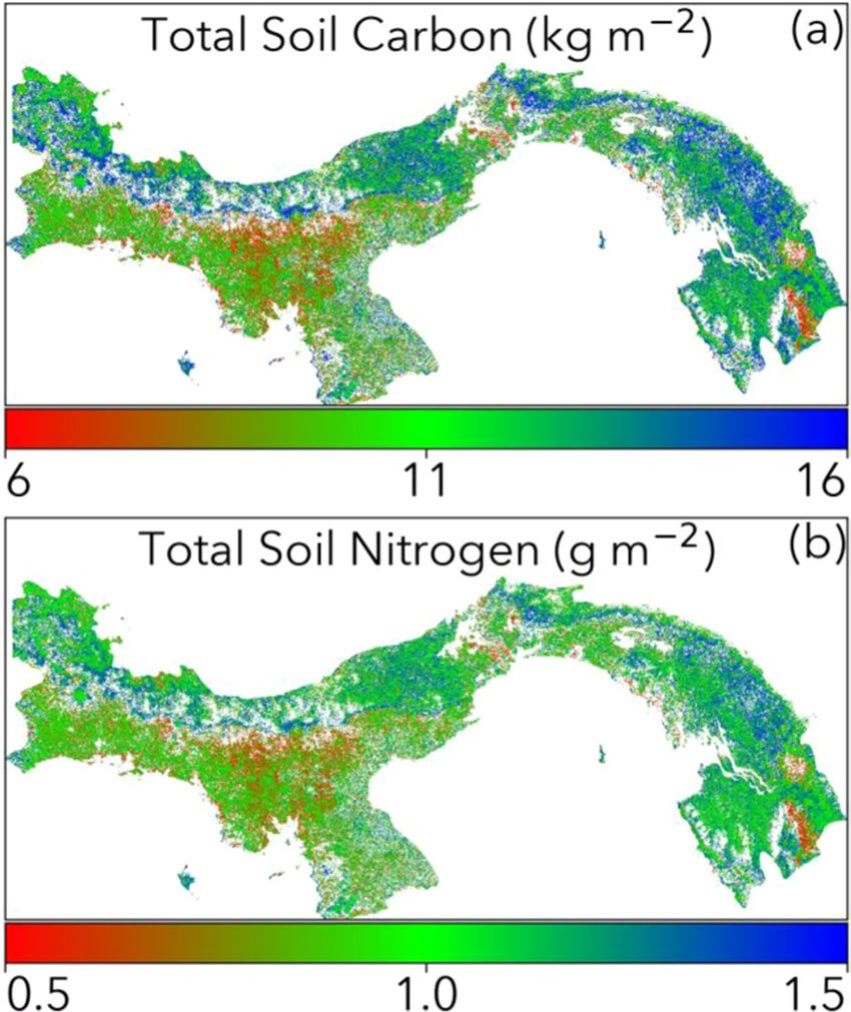
Panama-wide maps of (**a**) soil carbon and (**b**) nitrogen derived from plot-level relationships between soil measurements and satellite remotely sensed canopy properties. Reference uncertainty is 19% for soil carbon and 18% for soil nitrogen.

Given the significant coupling we found between *in situ* soil nutrients, soil C, and root biomass with remotely sensed canopy greenness limitation, we produced Panama-wide maps of soil C and N based on those respective relationships (Fig. 5). We note that the maps should be treated more as thought exercises than as definitive truth, relying both on assumptions about the representativeness of the plots to the region/country at large as well as the soil-canopy relationships. For example, some areas are inaccessible and not well sampled by the plots, and are unlikely to be representative of the actual soil nutrients. As a reference for uncertainty in the maps, the linear model RMSE divided by plot mean was 19% for soil carbon and 18% for soil N. Given these caveats, some interesting spatial patterns do emerge. Total soil C and N are relatively well-constrained, with maxima along the Caribbean side and minima along the Cordillera de Talamanca mountain range. Interestingly, these maps compare (qualitatively) similarly to existing soils maps for the country^109–111^. There is potential power in many of these maps, as tropical soil C, for example, represents a large uncertainty in global C cycle models^112,113^.

## Conclusions

We found significant coupling between remotely sensed canopy properties of greenness and productivity with *in situ* soil nutrients, C, and toxic elements for humid tropical forests across soil fertility and precipitation gradients. These patterns emerged across a two-fold increase in precipitation, but relationships of canopy properties with soil nutrients were as strong or stronger than relationships with precipitation, providing new insight into how we think about canopy scale assessments of tropical ecosystem fertility and productivity. Soil nutrients led to competing effects on canopy greenness, with P and cations leading to a decrease in greenness related to increases litterfall, likely related to decreased nutrient use efficiency. In contrast, soil N corresponded to increased canopy greenness, and no relationship with litterfall. Of particular interest was the use of a novel remote sensing index of canopy greenness limitation, which we found had strong relationships with soil N, soil C, and root biomass, highlighting the potential utility of a remote sensing product for assessing belowground nutrients and C storage in some tropical ecosystems. Future satellite missions using imaging spectroscopy, for example, may further refine these analyses, disentangling much of the canopy chemistry differences related to soil nutrient patterns^114,115^. These results may help explain interpretations of the greening of the tropics due to CO_2_ under nutrient limitations, and how climate projections and C cycle uncertainties are tied directly both to tropical ecosystem process understanding and soil carbon and nutrients.

## Supplementary information

Supplementary Information.
