# Supplementary material for: Competing effects of soil fertility and toxicity on tropical greening
Source: Sci Rep. 2020 Apr 21;10:6725. doi: 10.1038/s41598-020-63589-1 (PMC7174296; doi:10.1038/s41598-020-63589-1)
Supplement: Supplementary file 1 — Supplementary Information. [file 41598_2020_63589_MOESM1_ESM.pdf]

Supplementary Information

Competing effects of soil fertility and toxicity on tropical greening

Joshua B. Fisher[1,*], Naga V. Perakalapudi[2], Benjamin Turner[3], David S. Schimel[1], Daniela F. Cusack[3,4]

[1] Jet Propulsion Laboratory, California Institute of Technology, 4800 Oak Grove Drive, Pasadena, CA, 91109, USA

[2] Department of Astronautical Engineering, University of Southern California, 854 Downey Way, Los Angeles, CA, 90089, USA

[3] Smithsonian Tropical Research Institute, Apartado 0843-03092, Balboa, Ancon, Panama.

[4] Department of Ecosystem Science and Sustainability, Colorado State University, Campus Delivery 1476, Fort Collins, CO, 80523, USA

[*] *Corresponding author. E-mail: jbfisher@jpl.nasa.gov*

Author contributions: JBF and DFC formulated idea; JBF designed research; JBF and NVP performed research; DFC and BT provided data; all authors contributed to the writing of the paper.

The authors declare no conflict of interest.

Supplementary Information

Supplementary Table 1. All correlation coefficients and p-values for Figures 1-4.

		Raw (Black Points)		Soil Order (Red Points)	
		R^2	P	R^2	P
Figure 1	A	0.22	0.01	0.72	0.07
	B	0.05	0.17	0.85	0.02
	C	0.19	0.00	0.79	0.04
	D	0.14	0.01	0.77	0.05
	E	0.09	0.05	0.36	0.28
	F	0.14	0.01	0.01	0.85
Figure 2	A	0.37	0.11	0.94	0.01
	B	0.87	0.00	0.97	0.00
	C	0.87	0.00	0.97	0.00
	D	0.91	0.00	0.98	0.00
	E	0.83	0.00	0.88	0.02
	F	0.10	0.45	0.28	0.36
Figure 3		0.08	0.05	0.97	0.00
Figure 4	A	0.65	0.00	0.48	0.19
	B	0.64	0.00	0.71	0.07
	C	0.57	0.00	0.62	0.11

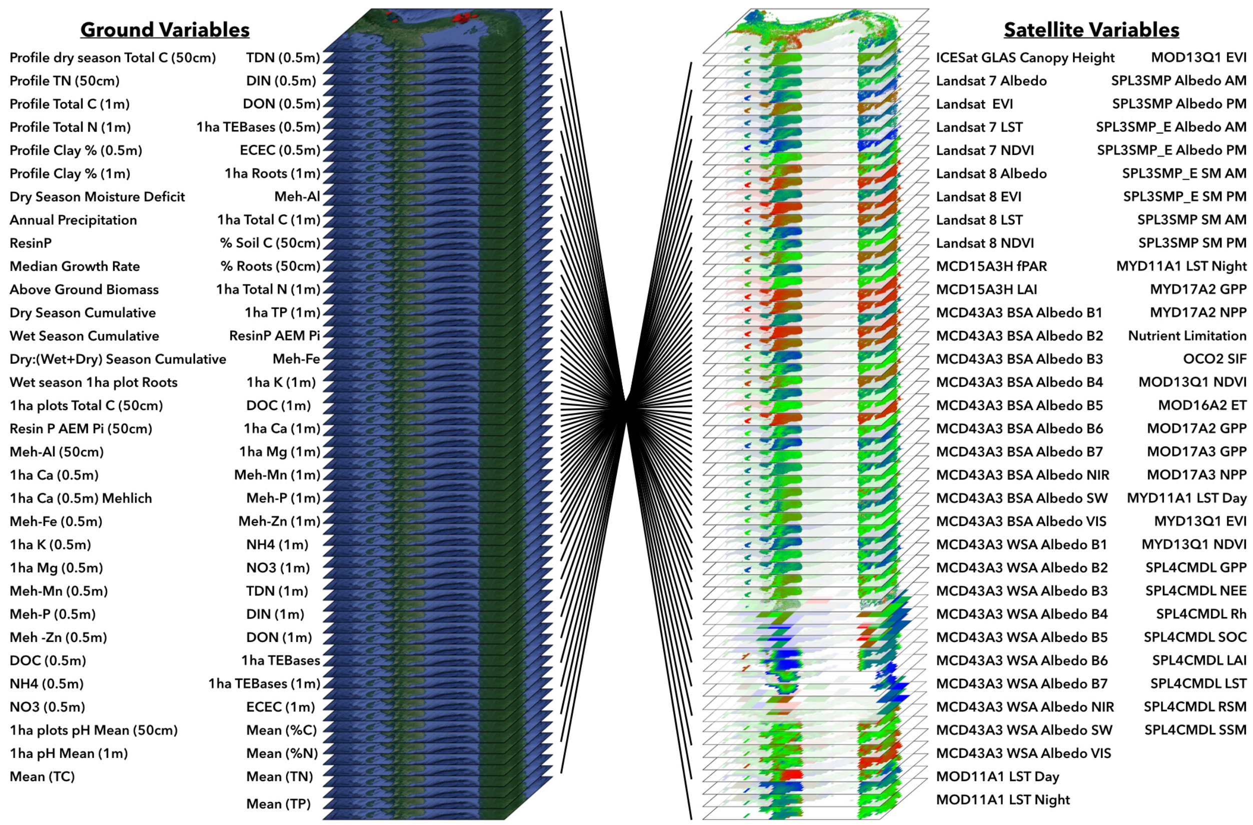

Supplementary Figure 1. This analysis combined among the most extensive single tropical forest soils datasets (>70 variables) with the most extensive satellite remote sensing compilation (>70 products) to date to assess linkages between canopy spectral properties and soil characteristics from 50 1-ha plots throughout Panama.

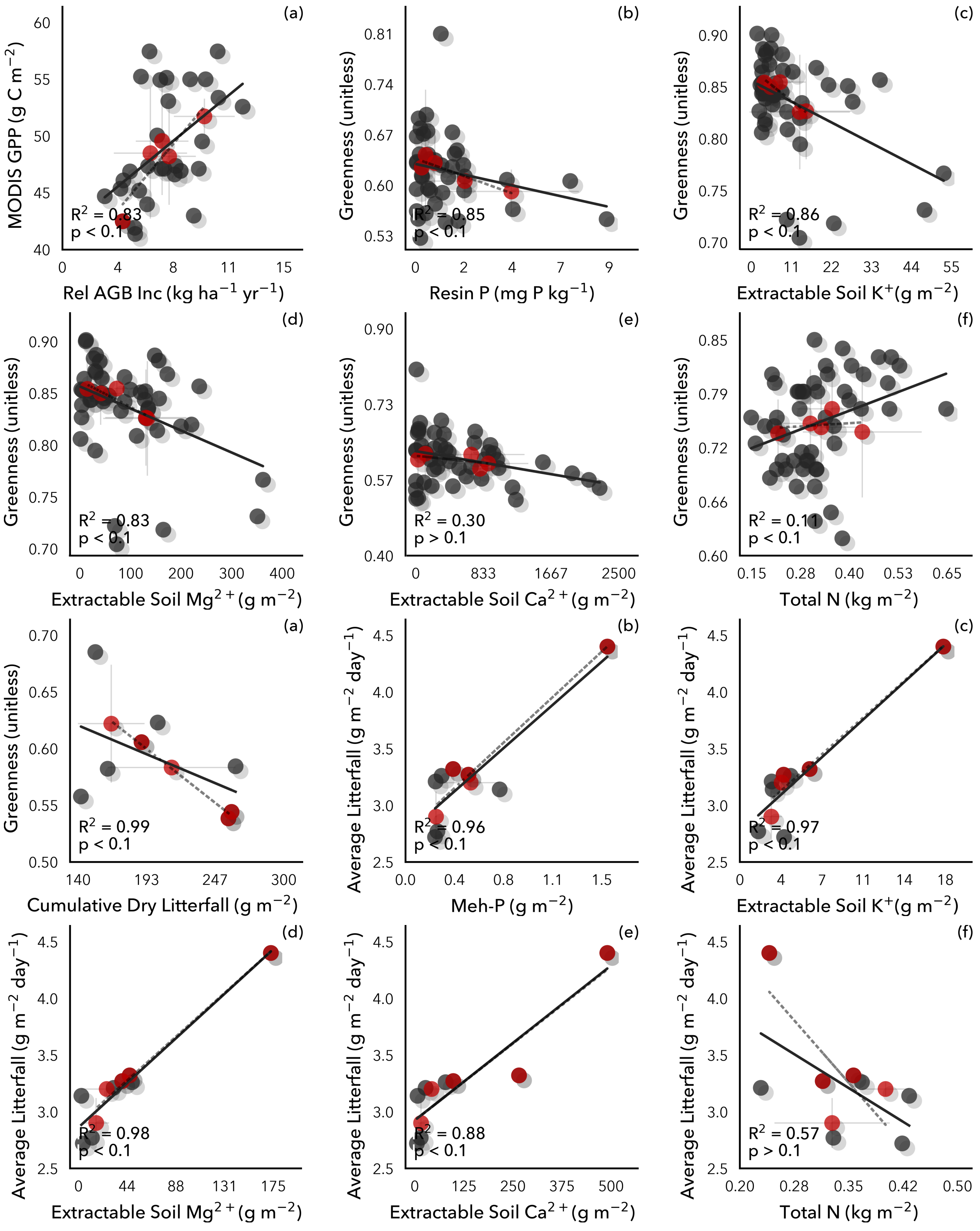
(a)
MODIS GPP (g C m^{-2})
Rel AGB Inc (kg ha^{-1} yr^{-1})
R^2 = 0.83
p < 0.1
(b)
Greenness (unitless)
Resin P (mg P kg^{-1})
R^2 = 0.85
p < 0.1
(c)
Greenness (unitless)
Extractable Soil K$^+$(g m^{-2})
R^2 = 0.86
p < 0.1
(d)
Greenness (unitless)
Extractable Soil Mg^{2+}(g m^{-2})
R^2 = 0.83
p < 0.1
(e)
Greenness (unitless)
Extractable Soil Ca^{2+}(g m^{-2})
R^2 = 0.30
p > 0.1
(f)
Greenness (unitless)
Total N (kg m^{-2})
R^2 = 0.11
p < 0.1
(a)
Greenness (unitless)
Cumulative Dry Litterfall (g m^{-2})
R^2 = 0.99
p < 0.1
(b)
Average Litterfall (g m^{-2} day^{-1})
Meh-P (g m^{-2})
R^2 = 0.96
p < 0.1
(c)
Average Litterfall (g m^{-2} day^{-1})
Extractable Soil K$^+$(g m^{-2})
R^2 = 0.97
p < 0.1
(d)
Average Litterfall (g m^{-2} day^{-1})
Extractable Soil Mg^{2+}(g m^{-2})
R^2 = 0.98
p < 0.1
(e)
Average Litterfall (g m^{-2} day^{-1})
Extractable Soil Ca^{2+}(g m^{-2})
R^2 = 0.88
p < 0.1
(f)
Average Litterfall (g m^{-2} day^{-1})
Total N (kg m^{-2})
R^2 = 0.57
p > 0.1

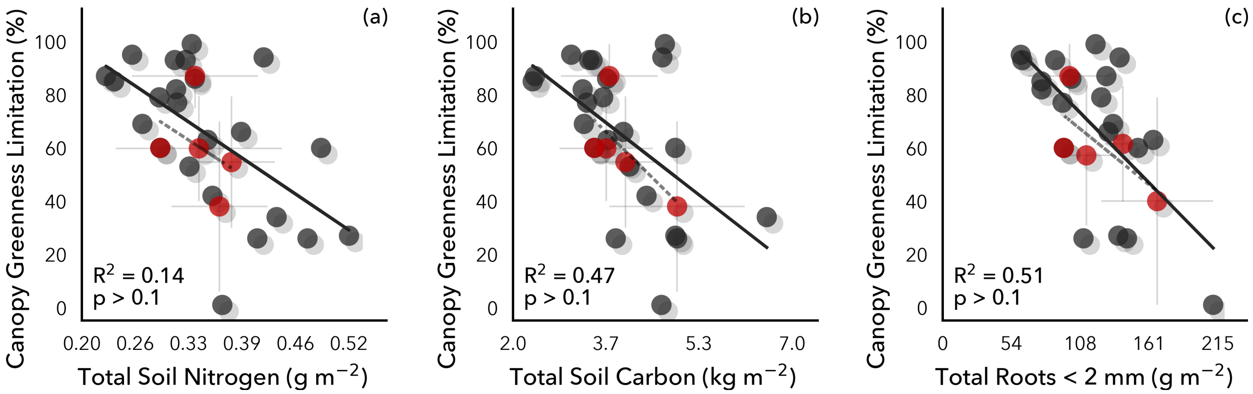

Supplementary Figure 2. As in Figures 1, 2, and 4, but all plots are normalized by mean annual precipitation.

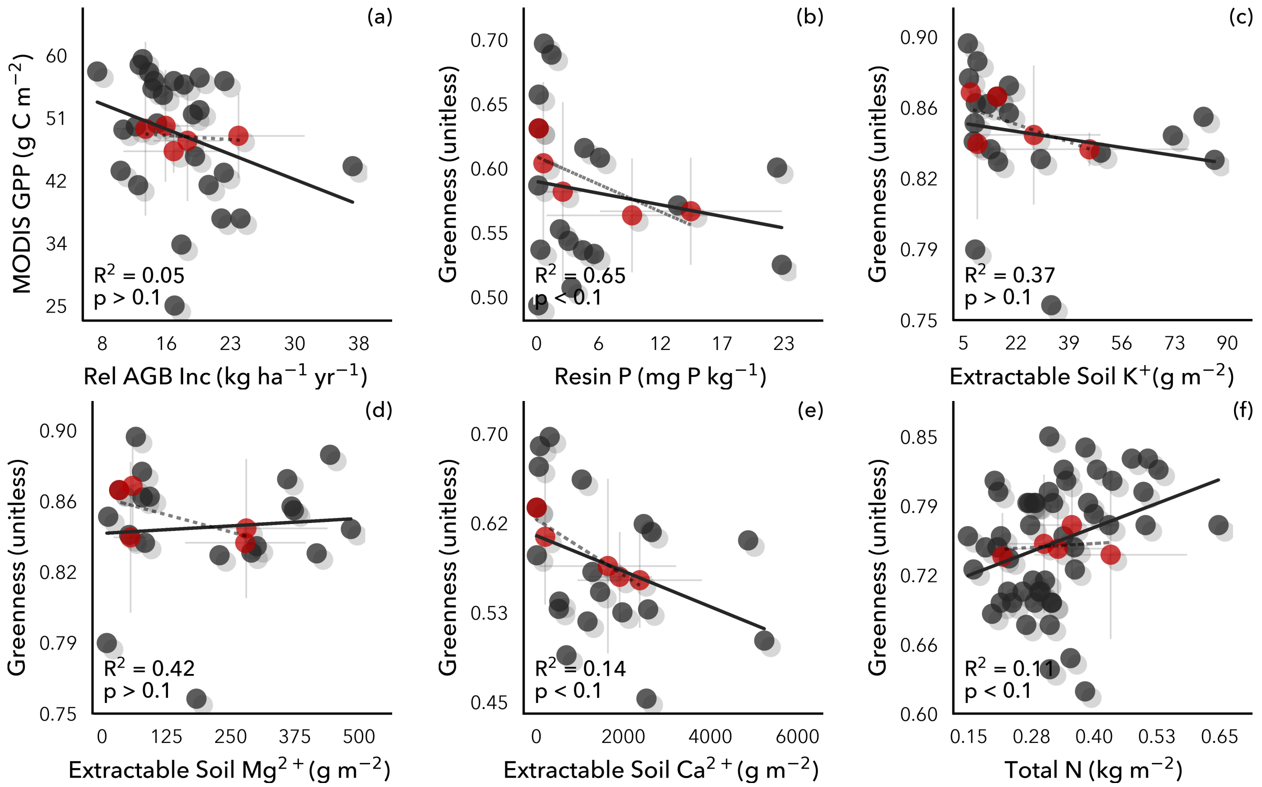
(a)
MODIS GPP (g C m^{-2})
60
51
42
34
25
R^2 = 0.05
p > 0.1
8
16
23
30
38
Rel AGB Inc (kg ha^{-1} yr^{-1})
(b)
Greenness (unitless)
0.70
0.65
0.60
0.55
0.50
R^2 = 0.65
p < 0.1
0
6
12
17
23
Resin P (mg P kg^{-1})
(c)
Greenness (unitless)
0.90
0.86
0.82
0.79
0.75
R^2 = 0.37
p > 0.1
5
22
39
56
73
90
Extractable Soil K$^+$(g m^{-2})
(d)
Greenness (unitless)
0.90
0.86
0.82
0.79
0.75
R^2 = 0.42
p > 0.1
0
125
250
375
500
Extractable Soil Mg^{2+}(g m^{-2})
(e)
Greenness (unitless)
0.70
0.62
0.53
0.45
R^2 = 0.14
p < 0.1
0
2000
4000
6000
Extractable Soil Ca^{2+}(g m^{-2})
(f)
Greenness (unitless)
0.85
0.79
0.72
0.66
0.60
R^2 = 0.11
p < 0.1
0.15
0.28
0.40
0.53
0.65
Total N (kg m^{-2})

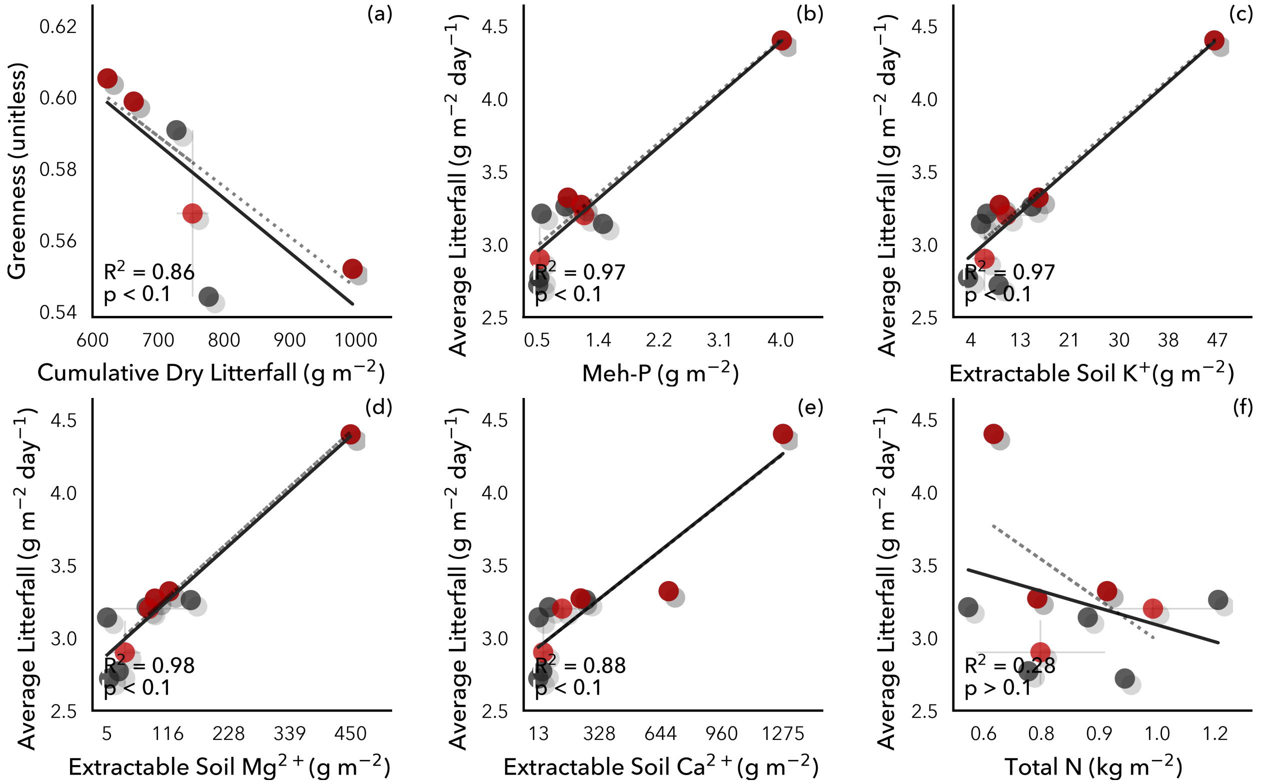
(a)
Greenness (unitless)
0.62
0.60
0.58
0.56
0.54
R^2 = 0.86
p < 0.1
600
700
800
900
1000
Cumulative Dry Litterfall (g m^{-2})
(b)
Average Litterfall (g m^{-2} day^{-1})
4.5
4.0
3.5
3.0
2.5
R^2 = 0.97
p < 0.1
0.5
1.4
2.2
3.1
4.0
Meh-P (g m^{-2})
(c)
Average Litterfall (g m^{-2} day^{-1})
R^2 = 0.97
p < 0.1
4
13
21
30
38
47
Extractable Soil K$^+$(g m^{-2})
(d)
Average Litterfall (g m^{-2} day^{-1})
R^2 = 0.98
p < 0.1
5
116
228
339
450
Extractable Soil Mg^{2+}(g m^{-2})
(e)
Average Litterfall (g m^{-2} day^{-1})
R^2 = 0.88
p < 0.1
13
328
644
960
1275
Extractable Soil Ca^{2+}(g m^{-2})
(f)
Average Litterfall (g m^{-2} day^{-1})
R^2 = 0.28
p > 0.1
0.6
0.8
0.9
1.0
1.2
Total N (kg m^{-2})

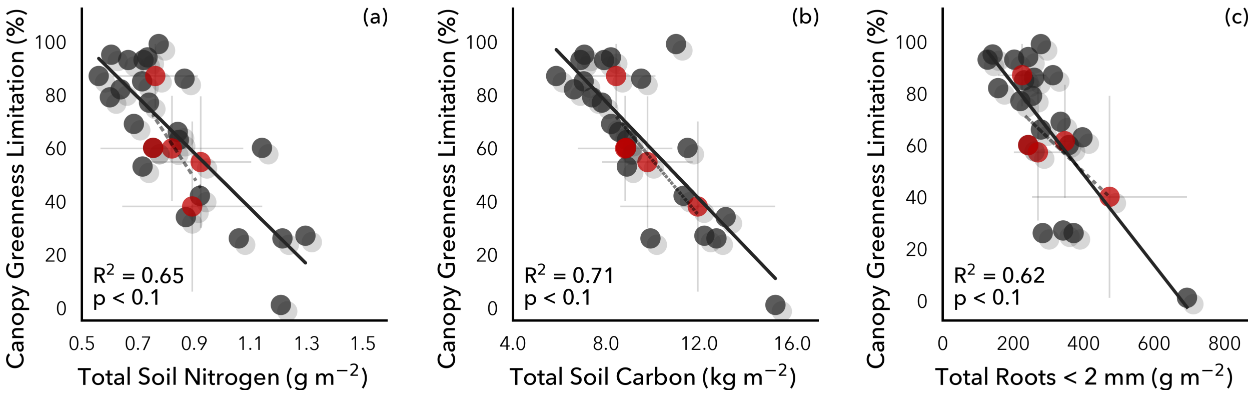

Supplementary Figure 3. As in Figures 1, 2, and 4, but using only wet season months (May – Dec). m – o remain unchanged due to the model requirement of a full year of data.

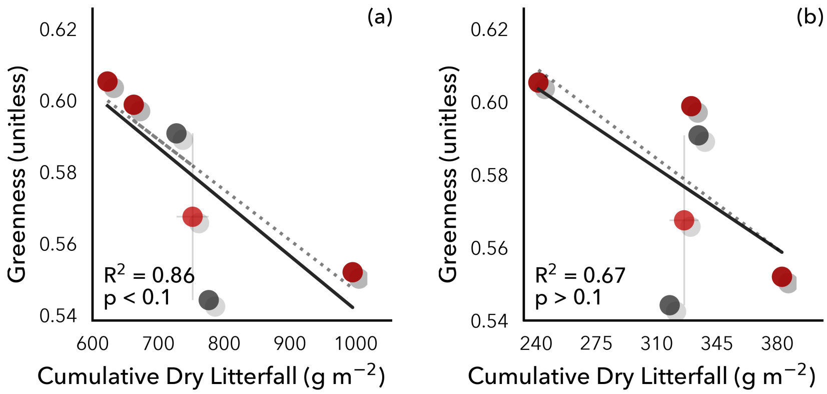

Supplementary Figure 4. Wet season cumulative litterfall versus albedo (MODIS black-sky) (a) original, and (b) normalized by precipitation.
